# Supplementary material for: HIVprotI: an integrated web based platform for prediction and design of HIV proteins inhibitors
Source: J Cheminform. 2018 Mar 9;10:12. doi: 10.1186/s13321-018-0266-y (PMC5845081; doi:10.1186/s13321-018-0266-y)
Supplement: Supplementary file 2 — Additional file 2. Source code of HIVProtI web server. [file 13321_2018_266_MOESM2_ESM.zip › HIVprotI_Source-code/hivprotI/weka-3-6-6/doc/weka/gui/package-summary.html]

weka.gui


---


|  |  |  |
| --- | --- | --- |
| **Overview** \| **Package** \| Class \| **Tree** \| **Deprecated** \| **Index** \| **Help** \| **Weka's home** | |  |
| **PREV PACKAGE**   **NEXT PACKAGE** | **FRAMES**    **NO FRAMES**     **All Classes** |  |


---

## Package weka.gui

| **Interface Summary** | |
| --- | --- |
| **CustomPanelSupplier** | An interface for objects that are capable of supplying their own custom GUI components. |
| **Logger** | Interface for objects that display log (permanent historical) and status (transient) messages. |
| **MainMenuExtension** | Classes implementing this interface will be displayed in the "Extensions" menu in the main GUI of Weka. |
| **TaskLogger** | Interface for objects that display log and display information on running tasks. |

| **Class Summary** | |
| --- | --- |
| **AttributeListPanel** | Creates a panel that displays the attributes contained in a set of instances, letting the user select a single attribute for inspection. |
| **AttributeSelectionPanel** | Creates a panel that displays the attributes contained in a set of instances, letting the user toggle whether each attribute is selected or not (eg: so that unselected attributes can be removed before classification). |
| **AttributeSummaryPanel** | This panel displays summary statistics about an attribute: name, type number/% of missing/unique values, number of distinct values. |
| **AttributeVisualizationPanel** | Creates a panel that shows a visualization of an attribute in a dataset. |
| **BrowserHelper** | A little helper class for browser related stuff. |
| **CheckBoxList** | An extended JList that contains CheckBoxes. |
| **ComponentHelper** | A helper class for some common tasks with Dialogs, Icons, etc. |
| **ConverterFileChooser** | A specialized JFileChooser that lists all available file Loaders and Savers. |
| **CostMatrixEditor** | Class for editing CostMatrix objects. |
| **DatabaseConnectionDialog** | A dialog to enter URL, username and password for a database connection. |
| **ExtensionFileFilter** | Provides a file filter for FileChoosers that accepts or rejects files based on their extension. |
| **FileEditor** | A PropertyEditor for File objects that lets the user select a file. |
| **GenericArrayEditor** | A PropertyEditor for arrays of objects that themselves have property editors. |
| **GenericObjectEditor** | A PropertyEditor for objects. |
| **GenericPropertiesCreator** | This class can generate the properties object that is normally loaded from the `GenericObjectEditor.props` file (= PROPERTY\_FILE). |
| **GUIChooser** | The main class for the Weka GUIChooser. |
| **GUIChooser.ChildFrameSDI** | Specialized JFrame class. |
| **HierarchyPropertyParser** | This class implements a parser to read properties that have a hierarchy(i.e. |
| **InstancesSummaryPanel** | This panel just displays relation name, number of instances, and number of attributes. |
| **JListHelper** | A helper class for JList GUI elements with DefaultListModel or derived models. |
| **JTableHelper** | A helper class for JTable, e.g. |
| **ListSelectorDialog** | A dialog to present the user with a list of items, that the user can make a selection from, or cancel the selection. |
| **Loader** | This class is for loading resources from a JAR archive. |
| **LogPanel** | This panel allows log and status messages to be posted. |
| **LogWindow** | Frame that shows the output from stdout and stderr. |
| **LookAndFeel** | A little helper class for setting the Look and Feel of the user interface. |
| **Main** | Menu-based GUI for Weka, replacement for the GUIChooser. |
| **Main.BackgroundDesktopPane** | DesktopPane with background image. |
| **Main.ChildFrameMDI** | Specialized JInternalFrame class. |
| **Main.ChildFrameSDI** | Specialized JFrame class. |
| **MemoryUsagePanel** | A panel for displaying the memory usage. |
| **Messages** | Messages. |
| **PropertyDialog** | Support for PropertyEditors with custom editors: puts the editor into a separate frame. |
| **PropertyPanel** | Support for drawing a property value in a component. |
| **PropertySelectorDialog** | Allows the user to select any (supported) property of an object, including properties that any of it's property values may have. |
| **PropertySheetPanel** | Displays a property sheet where (supported) properties of the target object may be edited. |
| **ResultHistoryPanel** | A component that accepts named stringbuffers and displays the name in a list box. |
| **ResultHistoryPanel.RKeyAdapter** | Extension of KeyAdapter that implements Serializable. |
| **ResultHistoryPanel.RMouseAdapter** | Extension of MouseAdapter that implements Serializable. |
| **SaveBuffer** | This class handles the saving of StringBuffers to files. |
| **SelectedTagEditor** | A PropertyEditor that uses tags, where the tags are obtained from a weka.core.SelectedTag object. |
| **SetInstancesPanel** | A panel that displays an instance summary for a set of instances and lets the user open a set of instances from either a file or URL. |
| **SimpleCLI** | Creates a very simple command line for invoking the main method of classes. |
| **SimpleCLIPanel** | Creates a very simple command line for invoking the main method of classes. |
| **SimpleCLIPanel.CommandlineCompletion** | A class for commandline completion of classnames. |
| **SimpleDateFormatEditor** | Class for editing SimpleDateFormat strings. |
| **SortedTableModel** | Represents a TableModel with sorting functionality. |
| **SortedTableModel.SortContainer** | Helper class for sorting the columns. |
| **SplashWindow** | A Splash window. |
| **SysErrLog** | This Logger just sends messages to System.err. |
| **ViewerDialog** | A downsized version of the ArffViewer, displaying only one Instances-Object. |
| **WekaTaskMonitor** | This panel records the number of weka tasks running and displays a simple bird animation while their are active tasks |

---


|  |  |  |
| --- | --- | --- |
| **Overview** \| **Package** \| Class \| **Tree** \| **Deprecated** \| **Index** \| **Help** \| **Weka's home** | |  |
| **PREV PACKAGE**   **NEXT PACKAGE** | **FRAMES**    **NO FRAMES**     **All Classes** |  |


---
